# Primary tumor location predicts poor clinical outcome with cetuximab in *RAS* wild-type metastatic colorectal cancer

**DOI:** 10.1186/s12876-017-0694-6

**Published:** 2017-11-23

**Authors:** Dalyong Kim, Sun Young Kim, Ji Sung Lee, Yong Sang Hong, Jeong Eun Kim, Kyu-pyo Kim, Jihun Kim, Se Jin Jang, Young-Kwang Yoon, Tae Won Kim

**Affiliations:** 1 Department of Oncology, Asan Medical Center, University of Ulsan College of Medicine, 88, Olympic-ro 43-gil, Songpa-gu, Seoul, 05505 Republic of Korea; 2 Clinical Research Center, Asan Institute for Life Sciences, Asan Medical Center, University of Ulsan College of Medicine, Seoul, South Korea; 3 Department of Pathology, Asan Medical Center, University of Ulsan College of Medicine, Seoul, South Korea; 4 Department of Hematology/Oncology, Yuseong Sun Hospital, 93, Bugyuseong-daero, Yuseong-gu, Daejeon, 34084 South Korea

**Keywords:** Primary tumor location, Metastatic colorectal cancer, Cetuximab, *RAS* wild-type, EGFR

## Abstract

**Background:**

In metastatic colorectal cancer, the location of the primary tumor has been suggested to have biological significance. In this study, we investigated whether primary tumor location affects cetuximab efficacy in patients with *RAS* wild-type metastatic colorectal cancer.

**Methods:**

Genotyping by the SequenomMassARRAY technology platform (OncoMap) targeting *KRAS*, *NRAS*, *PIK3CA*, and *BRAF* was performed in tumors from 307 patients who had been given cetuximab as salvage treatment. Tumors with mutated *RAS* (*KRAS* or *NRAS; n* = 127) and those with multiple primary location (*n* = 10) were excluded. Right colon cancer was defined as a tumor located in the proximal part to splenic flexure.

**Results:**

A total of 170 patients were included in the study (right versus left, 23 and 147, respectively). Patients with right colon cancer showed more mutated *BRAF* (39.1% vs. 5.4%), mutated *PIK3CA* (13% vs. 1.4%), poorly differentiated tumor (17.4% vs. 3.4%), and peritoneal involvement (26.1% vs. 8.8%) than those with left colon and rectal cancer. Right colon cancer showed poorer progression-free survival (2.0 vs.5.0 months, *P* = 0.002) and overall survival (4.1 months and 13.0 months, *P* < 0.001) than the left colon and rectal cancer. By multivariable analysis, *BRAF* mutation, right colon primary, poorly differentiated histology, and peritoneal involvement were associated with risk of death.

**Conclusions:**

In *RAS* wild-type colon cancer treated with cetuximab as salvage treatment, right colon primary was associated with poorer survival outcomes than left colon and rectal cancer.

## Background

Colorectal cancer (CRC) represents 10% of cancer incidence globally, and it is the fourth leading cause of cancer-related deaths worldwide. South Korea has one of the highest incidences of CRC in the world [1]. The survival of metastatic CRC has gradually been improved with advancements in medical therapy, which include not only the development of new drugs but also the discovery of predictive biomarkers.

Previous studies have suggested that primary tumor location (PTL) may be a surrogate for tumor biology that may affect treatment outcomes [2, 3]. In terms of embryology and molecular carcinogenesis, CRC can be divided into distinct disease entities according to the PTL; the right side of the colon, including the cecum, the ascending colon, and the transverse colon is derived from the midgut, while the remaining parts of the colon and rectum come from the hindgut [2]. Tumors of the right colon (RC) tend to more frequently exhibit a poorly differentiated histology, *BRAF* mutation, a hypermethylated phenotype, and microsatellite instability (MSI), while *c-MYC* expression occurs more commonly in tumors of left colon and rectum(LC) than in those in the RC [2, 4, 5].

The prognostic and predictive implications of PTL have been addressed in numerous studies, but there is no clear consensus on the role of PTL in treatment decisions. Generally, RC cancer is associated with poorer survival compared to LC cancer [6, 7], and recent evidences have shown that patients with RC cancer may respond poorly to anti-epidermal growth factor receptor (EGFR) antibodies, such as cetuximab or panitumumab, which are the backbone of treatment for metastatic CRC [8, 9]. However, the predictive value of PTL should be analyzed in consideration of *RAS* mutation,the most powerful predictor of response of anti-EGFR antibodies [10, 11].

In our current study, we investigated the association between PTL and clinical outcomes in *RAS* wild-type metastatic CRC patients who received cetuximab as a salvage treatment.

## Methods

### Patients

**Fig. 1:**
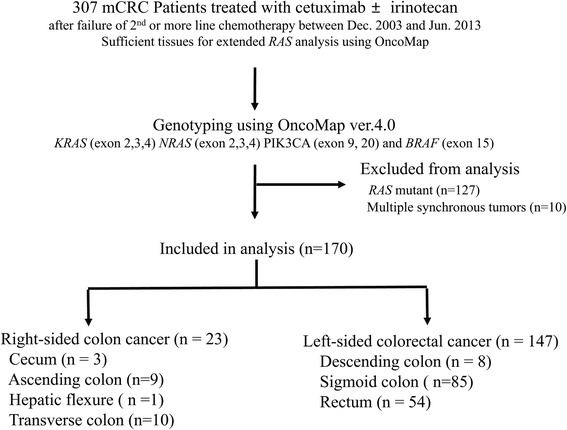
Flow diagram for the patient selection process. mCRC, metastatic colorectal cancer

We retrospectively reviewed the medical records of 307 metastatic CRC patients treated with cetuximab with or without irinotecan as a salvage treatment between December 2003 and June 2013 at Asan Medical Center, Seoul, Republic of Korea. All of them were given cetuximab as a third or later line of treatment for metastatic CRC and had sufficient tissue to conduct extended *RAS* analyses. After extended *RAS* testing, 127 *RAS* mutant patients were excluded from the analysis, and additional 10 patients with synchronous multiple colon cancers were excluded due to an inability to define PTL. Finally, 170 patients were analyzed and assigned to either RC or LC group (Fig. 1). Clinicopathologic variables, including age, gender, initial stage, histologic differentiation, metastatic sites, MSI status (determined as previously described [12] by the Bethesda panel), and details of treatment given before cetuximab were extracted from medical records. This study was conducted in accordance with the declaration of Helsinki and was approved by the Institutional Review Board of Asan Medical Center. Informed consent was obtained in all participants except patients who were dead at the time of this study. Institutional Review Board of Asan Medical Center approved to waive the requirement to obtain informed consent from the dead according to Bioethics and Biosafety Act in Korea.

### Genotyping

Mutational analysis was done using the SequenomMassARRAY technology platform (OncoMap version 4.0), as previously described [13, 14]. A pathologist (JK) reviewed formalin-fixed paraffin-embedded tissue and marked tumor portions, where DNA was extracted from using the QIAamp DNA Tissue kit (Qiagen, Hilden, Germany) according to the manufacturer’s instructions. Multiplex polymerase chain reaction amplification using iPLEXchemistry (#10134–2; Sequenom, San Diego, CA), and homogenous mass extension validation of mutation were implemented. Single-base extension was done and a MALDI-TOF mass spectrometer was used to determine the unique mass value according to the mutation site. Target genes were *KRAS* (exon 2, 3, and 4), *NRAS* (exon 2, 3, and 4), *PIK3CA* (exon 9 and 20), and *BRAF* (exon 15).

### Tumor locations

The tumor location was determined based on operation records and colonoscopy findings. The RC group consisted of cancers occurring in the cecum, the ascending colon, hepatic flexure, and the transverse colon, while the LC group included primary tumors from the splenic flexure to the rectum.

### Statistical analysis

The statistical tests were exploratory in nature. Demographic and baseline clinical characteristics were compared according to the PTL. Fisher’s exact test and the Mann–Whitney U-test were used for categorical variables and continuous variables, respectively. The Kaplan–Meier method was used to estimate progression-free survival (PFS) and overall survival (OS), and the survival outcomes were compared according to PTL by the log-rank test. PFS was defined as the time from the date of the first administration of cetuximab to the date of documented disease progression or the date of death from any cause, if progression was not documented before death. OS was defined as the time from the date of first cetuximab dose to the date of death from any cause. To evaluate clinical outcomes by PTL, the Cox proportional hazards regression model was used for multivariable analysis. In these analyses, we selected statistically significant or clinically meaningful variables as predictors for the final model. Thus, PFS and OS were adjusted for age, sex, presence of peritoneal seeding, histologic grade, and *BRAF* mutational status. Hazard ratio (HR) associated with each variable was suggested with 95% confidence interval (CI). All statistical analyses were two-sided, with a level of significance established at *p* < 0.05, and performed using the Statistical Package for Social Sciences version 21.0 (IBM Corp., Armonk, NY). The Kaplan–Meier curves were drawn using STATA software (StataCorp. 2015. *Stata Statistical Software: Release 14*. College Station, TX: StataCorp LP).

### Role of the funding source

The study funders had no role in the design, analysis, interpretation or manuscript preparation. All authors prepared the drafts of this report and approved the submission. The corresponding author had full access to all the data and final responsibility to submit for publication.

## Results

### Patient characteristics

Among a total of 170 *RAS* wild-type patients, 23 were classified as belonging to the RC group and 147 as belonging to the LC group (Fig. 1). The baseline characteristics of the study patients are presented in Table 1. All patients were treated with cetuximab as third or later-line therapy, and half of them received it in combination with irinotecan. All patients were treated with irinotecan before cetuximab; no significant difference was seen in previously exposed chemotherapeutic agents (oxaliplatin, fluoropyrimidine, and bevacizumab). The median age was similar between the groups, while the proportion of female patients was higher in the RC group, without statistical significance. The most common metastatic organ was liver in both groups, while peritoneal metastasis was more common in RC cancer than in LC cancer (26.1% vs. 8.8%, *p* = 0.026). The proportion of unfavorable histology, including poorly differentiated adenocarcinoma and signet ring cell carcinoma, was 20.0% in RC and 5.0% in LC (*p* = 0.031). The interval between first-line chemotherapy and cetuximab treatment was slightly shorter in the RC group than the LC group, without statistical significance (16.4 vs. 18.2 months, *p* = 0.552).

**Table 1:** Baseline characteristics according to primary tumor location

Characteristics	Right colon (*n* = 23)	Left colon and rectum (*n* = 147)	*P*-value*
Age (years)			0.546
Median (range)	57 (36–72)	55 (24–74)	
Gender			0.154
Female	10 (43.5%)	42 (28.6%)	
Male	13 (56.5%)	105 (71.4%)	
Stage at presentation			0.292
Stage IV	20 (87.0%)	110 (74.8%)	
Stage I-III	3 (13.0%)	37 (25.2%)	
Histology			0.031
W/D or M/D	16 (80.0%)	136 (95.0%)	
P/D or SRCC	4 (20.0%)	7 (5.0%)	
Number of metastasized organs			>0.999
1	13 (56.5%)	81 (55.1%)	
≥ 2	10 (43.5%)	66 (44.9%)	
Metastasized organ			
Liver	12 (52.2%)	106 (72.1%)	0.086
Lung	7 (30.4%)	65 (44.2%)	0.260
Peritoneum	6 (26.1%)	13 (8.8%)	0.026
Treatment lines			0.921
3	16 (69.6%)	104 (70.7%)	
4	6 (26.1%)	35 (23.8%)	
≥5	1 (4.3%)	8 (5.4%)	
Prior treatment			
Oxaliplatin	23 (100%)	144 (98.0%)	1.00
Bevacizumab	7 (30.4%)	25 (17.0%)	0.151
5-fluoropyrimidine	20 (87.0%)	136 (92.5%)	0.408
Regimen			0.482
Cetuximab + irinotecan	13 (56.5%)	97 (66.0%)	
Cetuximab monotherapy	10 (43.5%)	50 (34.0%)	
*BRAF*			<0.001
Wild-type	14 (60.9%)	139 (94.6%)	
Mutant	9 (39.1%)	8 (5.4%)	
*PIK3CA*			0.018
Wild-type	20 (87.0%)	145 (98.6%)	
Mutant	3 (13.0%)	2 (1.4%)	
MSI status			0.156
MSS	19 (82.6%)	85 (57.8%)	
MSI-low	0 (0%)	3 (2.0%)	
MSI-high	0 (0%)	1 (0.7%)	
Not checked	4 (17.4%)	58 (39.5%)	

Abbreviations: *W/D* well-differentiated, *M/D* moderately differentiated, *P/D* poorly differentiated, *SRCC* signet ring cell carcinoma, *MSI* microsatellite instability, *MSS* microsatellite stable

^*^ *P*-value by Fisher’s exact test or Mann-Whitney U-test as appropriate

### Molecular information according to PTL

The results of genotyping are summarized in Table 1. The *BRAF* V600E mutation was detected in nine (39.1%) patients in the RC group and eight (5.4%) in the LC group. The *PIK3CA *mutation was also more frequently detected in the RC group (13% vs. 1.4%, *p* = 0.018). The MSI test is not routinely performed for metastatic CRC in our institution, so information for MSI status was only available for 108 patients (63.5%), among whom only 1 patient, in the LC group, had an MSI-high tumor.

### Clinical outcomes by primary tumor location

**Fig. 2:**
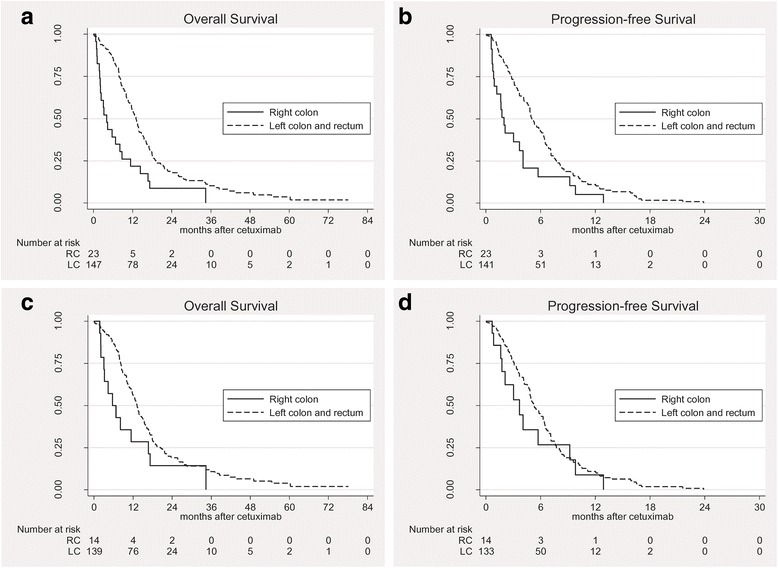
Kaplan–Meier estimates of (**a**) overall survival and (**b**) progression-free survival according to primary tumor location in *RAS* wild-type metastatic colorectal cancer patients (*n* = 170), and (**c**) overall survival and (**d**) progression-free survival in *RAS* and *BRAF* wild-type patients (*n* = 153)

A profound difference in OS according to PTL was observed: 4.1 months (95% CI, 2.1 to 8.1) in the RC group and 13.0 months (95% CI 11.5 to 14.0) in the LC group (*p <* 0.001) (Fig. 2a). The PFS was also poorer in the RC group (2.0 months, 95% CI 0.9 to 4.0) than in the LC group (5.0 months, 95% CI 4.5 to 6.3, *p* = 0.002) (Fig. 2b).

*BRAF* mutation was significantly associated with poor clinical outcome: OS in *BRAF* wild-type and mutants were 13.0 months and 4.1 months (*p* < 0.0001), respectively. PFS was 5.1 months in *BRAF* wild-type, and 1.2 months in *BRAF* mutants (*p* < 0.0001).

Within the *RAS* and *BRAF* wild-type population (*n* = 153, 14 in RC group and 139 in LC group), OS was still poorer in the RC group than in the LC group (5.7 months vs. 13.2 months, respectively), with marginal significance (*p* = 0.055, Fig. 2c). PFS did not significantly differ between the two groups when *BRAF* mutants were excluded (3.7 months in RC group vs. 5.3 months in LC group, *p* = 0.219; Fig. 2d).

The presence of peritoneal metastasis, *BRAF* mutation, unfavorable histology (poorly differentiated adenocarcinoma or signet ring cell carcinoma), and PTL were statistically meaningful parameters in bivariate analysis for OS and PFS using the Cox proportional hazard model (Tables 2 and 3). By multivariable analysis, RC was associated with a poorer OS (hazards ratio 1.84, 95% CI 1.10 to 3.09, *p* = 0.021, Table 2), and there was a trend toward a lower PFS (HR 1.55, 95% CI 0.92 to 2.61, *p* = 0.099, Table 3).

**Table 2:** Bivariate and multivariable analysis for overall survival

Characteristic	Crude HR (95% CI)	*P*-value* (bivariate^a^)	Adjusted HR (95% CI)	*P*-value* (multivariable^a^)
Primary tumor location				
Right vs. Left	2.29 (1.46–3.62)	<0.001	1.84 (1.10–3.09)	0.021
Age				
>60 years vs. ≤ 60	1.06 (0.76–1.47)	0.750	1.07 (0.75–1.51)	0.720
Gender				
Female vs. Male	0.92(0.65–1.30)	0.623	0.97 (0.66–1.40)	0.852
Stage at presentation				
Stage IV vs. Stage I-III	1.12 (0.77–1.63)	0.554		
Histologic grade				
P/D or SRC vs. W/D or M/D	4.35 (2.21–8.57)	<0.001	3.08 (1.49–6.34)	0.002
Presence of liver metastasis				
Yes vs. No	1.29 (0.91–1.82)	0.156		
Presence of lung metastasis				
Yes vs. No	0.84 (0.61–1.15)	0.279		
Presence of peritoneal metastasis				
Yes vs. No	2.91 (1.79–4.75)	<0.001	2.05 (1.17–3.60)	0.013
Number of metastasized organs				
≥ 2 vs. 1	1.10 (0.80–1.52)	0.540		
*BRAF*				
Mutant vs. Wild-type	3.24 (1.94–5.43)	<0.001	2.84 (1.60–5.03)	<0.001
*PIK3CA*				
Mutant vs. Wild-type	1.97 (0.80–4.84)	0.138		

Abbreviations: *HR* hazard ratio, *CI* confidence interval, *P/D* poorly differentiated, *SRCC* signet ring cell carcinoma, *W/D* well-differentiated, *M/D* moderately differentiated

^*^ *P*-value by Cox’s proportional hazards regression

^a^Clinically meaningful variables and those with *p* < 0.05 by bivariate analysis were entered into the multivariable analysis model

**Table 3:** Bivariate and multivariable analysis for progression-free survival

Characteristic	Crude HR (95% CI)	*P*-value* (bivariate^a^)	Adjusted HR (95% CI)	*P*-value* (multivariable^a^)
Primary tumor location				
Right vs. Left	2.09 (1.31–3.33)	0.002	1.55 (0.92–2.61)	0.099
Age				
>60 years vs. ≤ 60	0.77 (0.54–1.08)	0.133	0.77 (0.53–1.13)	0.180
Gender				
Female vs. Male	1.15 (0.80–1.64)	0.449	1.11 (0.75–1.64)	0.616
Stage at presentation				
Stage IV vs. Stage I-III	0.99 (0.66–1.49)	0.965		
Histologic grade				
P/D or SRC vs. W/D or M/D	5.37(2.24–12.87)	<0.001	3.06 (1.22–7.67)	0.017
Presence of liver metastasis				
Yes vs. No	1.04 (0.73–1.50)	0.816		
Presence of lung metastasis				
Yes vs. No	0.87 (0.63–1.21)	0.419		
Presence of peritoneal metastasis				
Yes vs. No	2.53 (1.53–4.19)	<0.001	2.08 (1.19–3.63)	0.010
Number of metastasized organs				
≥ 2 vs. 1	1.11 (0.80–1.53)	0.543		
*BRAF*				
Mutant vs. Wild-type	3.56 (2.11–6.01)	<0.001	3.07 (1.73–5.46)	<0.001
*PIK3CA*				
Mutant vs. Wild-type	2.60 (0.95–7.11)	0.062		

Abbreviations: *HR* hazard ratio, *CI* confidence interval, *P/D* poorly differentiated, *SRCC* signet ring cell carcinoma, *W/D* well-differentiated, *M/D* moderately differentiated

^*^P-value by Cox’s proportional hazards regression

^a^Clinically meaningful variables and those with *p* < 0.05 by bivariate analysis were entered into the multivariable analysis model

## Discussion

We observed in our current analysis that RC was associated with poor OS and PFS outcomes in *RAS* wild-type metastatic CRC patients treated with cetuximab as salvage therapy. OS was still poorer in the RC group when *BRAF* mutants were excluded. These findings suggest that PTL may be a factor in deciding on salvage chemotherapy with cetuximab in *RAS* wild-type patients.

Several studies have suggested that clinical benefits from anti-EGFR treatment may differ according to PTL. In data from a phase II trial of cetuximab-based chemotherapy, LC cancer was found to be associated with significantly longer PFS and OS than RC cancer in *KRAS* exon 2 wild-type patients [9]. Similarly, the NCIC CO.17 trial, a phase III study that compared cetuximab with the best supportive care in refractory metastatic CRC, showed that patients with LC cancer had benefit from cetuximab in terms of PFS, but those with RC cancer did not in *KRAS* exon 2 wild-type population [8]. Post-hoc analysis of the CALGB 80405 trial, a phase III trial that compared cetuximab and bevacizumab in front-line settings, also showed that cetuximab-treated *KRAS* exon 2 wild-type patients with RC cancer had markedly poorer survival than those with LC cancer (16.7 months in RC and 36.0 in LC, *p* < 0.0001) [15]. All of these analyses were performed in *KRAS* exon 2 wild-type patients, so the possibility that *RAS* mutations other than *KRAS* exon 2 may have contributed to the relatively poorer outcome in RC cancer in these studies could not be excluded. Recent studies for *RAS* wild-type subgroup of randomized trials which compared anti-EGFR treatment with chemotherapy +/− bevacizumab addressed this issue, by showing OS, PFS and objective response rate with anti-EGFR treatment was poorer in RC cancer than in LC cancer [16, 17].

A strength of our present study is that we excluded all *RAS* mutants from our analysis using a highly sensitive high-throughput method, the SequenomMassARRAY system. Our previous study showed that this method was more helpful for selecting candidates for cetuximab treatment than less-sensitive Sanger sequencing [14]. This means that the poorer outcomes with anti-EGFR treatment in RC cancer than in LC cancer were maintained even after all *RAS* mutants with low-allele frequency were excluded.

We further found in our analysis that *BRAF* and *PIK3CA* mutants were enriched in RC cancer, as shown in previous studies [5], which may partly explain the different therapeutic effects of anti-EGFR according to the PTL. However, the difference in OS was still retained, even after excluding *BRAF* mutants. Although we should be careful interpreting this subset analysis due to small sample size and relatively low proportion of RC (in *RAS* and *BRAF* wild-type subgroup, *n* = 14 for RC compared to *n* = 139 in LC), PTL may be associated with poor prognostic biological features other than *RAS* or *BRAF* mutation. The prognostic impact of PTL irrespective of *RAS* and *BRAF* mutation was also suggested by subgroup analyses of 3 randomized trials on anti-EGFR vs. chemotherapy +/− bevacizumab (FIRE3, PRIME, and PEAK), which showed poor prognosis of RC in patients with *RAS/BRAF* wild-type metastatic CRC [18, 19].

Recent studies have suggested that biologic features of RC cancer that may explain the poor prognosis or resistance to anti-EGFR treatment. A translational study in PETACC-3 trial reported that *BRAF*-mutant-like tumors, which were *BRAF* wild-type but shared similar gene expression profile with *BRAF* mutant tumors, were enriched in RC and had poor prognosis [20]. The abundant expression of epiregulin and amphiregulin, which are known to be predictors of good response to anti-EGFR treatment, was shown to be more prevalent in LC cancer than in RC cancer [21]. On the other hand, MiR-31-3p, a poor predictor for anti-EGFR response, was shown to be overexpressed more frequently in RC cancer than in LC cancer [22]. In addition, Consensus Molecular Subtype (CMS)1, which is associated with a poorer survival rate after relapse, is more common in RC [23]. However, there might be more unknown features of biologic relevance of PTL, since recent translational study from CALGB 80405 showed PTL was independent prognostic factor when adjusted to *BRAF* mutation, MSI and CMS [24].

It is uncertain whether PTL is just a negative prognostic indicator reflecting tumor biology irrespective of treatment or is a genuine predictive marker of the response to anti-EGFR treatment. In CALGB 80405 trial, the OS was better in LC than in RC cancer in bevacizumab-treated patients, although the interaction between the PTL and the treatment (cetuximab versus bevacizumab) was significant (*p* = 0.005) [15]. This prognostic as well as predictive impact of PTL was consistently shown by the pooled analysis of 6 randomized trials (5 in first-line and 1 in second-line setting) on anti-EGFR in terms of OS and PFS although the interaction between sidedness and treatment effect was not always significant in all of the trials [16]. However, in NCIC CO.17 study, a randomized study in chemo-refractory setting, PFS and OS in the best supportive care arm was not affected by PTL, implying that it was not prognostic, but only predictive of PFS benefit from cetuximab (interaction *p* = 0.002) [8]. To date, collective evidences are suggesting high likelihood of no clinical benefit from anti-EGFR in RC cancer, at least in first-line setting, and possibly in later-line treatment. PTL seems to be also prognostic for metastatic CRC in first-line setting, but unclear in chemo-refractory patients.

In our current study, PTL showed a significant relationship with OS in multivariable analysis, but not in PFS; the prognostic impact of PTL seems to be more prominent than the predictive role in these results. However, the limitations of our present analysis hamper such an interpretation; the sample size of our patients with RC cancer was too small and we did not include a control group without cetuximab. Thus, it was difficult to conclude whether PTL is purely prognostic or predictive from our present study.

The characteristics of our study patients were comparable to those of previous reports, namely frequent poorly differentiated histology and peritoneal seeding, as well as the mutation profile previously mentioned [2, 6, 25]. However, the proportion of RC patients in our study population was relatively too small (13.5%, 23/170) to have adequate power of comparison according to PTL. This probably resides in the low proportion of proximal colon cancer in Korean populations: 18.6% in male and 24.2% in female according to 2009 statistics from national registry [26]. This is consistent with the racial difference in subsite-specific CRC incidence in United States; Asia-Pacific islanders had lowest proportion of proximal colon cancer (28% in male, 34% in female) compared to other racial groups (usually ≥40%) [27]. The association of racial difference in subsite distribution and prognosis according to treatment warrants further investigation.

Our current study had other limitations, including its retrospective nature, the heterogeneous regimen of cetuximab, insufficient information on MSI, and the lack of biological information other than hotspot mutation profiles, such as gene expression or the CpG island methylation profile.

## Conclusions

PTL in RC might be associated with a poor OS outcome in patients with *RAS* wild-type metastatic CRC being treated with cetuximab as salvage treatment. Despite of the limitations from the retrospective study of small sample size, this finding supports the conclusions of previous studies that PTL may be a surrogate for tumor biology and should be considered as a biomarker and as a stratification factor in conducting clinical trials. Further studies are needed to validate the impact of PTL on treatment outcomes in various clinical settings.

## Abbreviations

CI: Confidence interval
CRC: Colorectal cancer
EGFR: Epidermal growth factor receptor
HR: Hazard ratio
LC: Left colon and rectum
MSI: Microsatellite instability
OS: Overall survival
PFS: Progression-free survival
PTL: Primary tumor location
RC: Right colon
